# S-540956, a CpG Oligonucleotide Annealed to a Complementary Strand With an Amphiphilic Chain Unit, Acts as a Potent Cancer Vaccine Adjuvant by Targeting Draining Lymph Nodes

**DOI:** 10.3389/fimmu.2021.803090

**Published:** 2021-12-23

**Authors:** Takayuki Nakagawa, Tetsuya Tanino, Motoyasu Onishi, Soichi Tofukuji, Takayuki Kanazawa, Yukichi Ishioka, Takeshi Itoh, Akira Kugimiya, Kazufumi Katayama, Takuya Yamamoto, Morio Nagira, Ken J. Ishii

**Affiliations:** ^1^ Pharmaceutical Research Division, Shionogi & Co., Ltd., Osaka, Japan; ^2^ Laboratory of Adjuvant Innovation, Center for Vaccine and Adjuvant Research (CVAR), National Institute of Biomedical Innovation, Health and Nutrition (NIBIOHN), Osaka, Japan; ^3^ Laboratory of Mock-up Vaccine Project, Center for Vaccine and Adjuvant Research (CVAR), National Institute of Biomedical Innovation, Health and Nutrition (NIBIOHN), Osaka, Japan; ^4^ Division of Vaccine Science, Department of Microbiology and Immunology, The Institute of Medical Science, The University of Tokyo (IMSUT), Tokyo, Japan

**Keywords:** cancer peptide vaccine, adjuvant, CpG oligonucleotide, delivery system, draining lymph node

## Abstract

Robust induction of cancer-antigen-specific CD8^+^ T cells is essential for the success of cancer peptide vaccines, which are composed of a peptide derived from a cancer-specific antigen and an immune-potentiating adjuvant, such as a Toll-like receptor (TLR) agonist. Efficient delivery of a vaccine antigen and an adjuvant to antigen-presenting cells in the draining lymph nodes (LNs) holds key to maximize vaccine efficacy. Here, we developed S-540956, a novel TLR9-agonistic adjuvant consisting of B-type CpG ODN2006 (also known as CpG7909), annealed to its complementary sequence oligodeoxynucleotide (ODN) conjugated to a lipid; it could target both a cancer peptide antigen and a CpG-adjuvant in the draining LNs. S-540956 accumulation in the draining LNs and activation of plasmacytoid dendritic cells (pDCs) were significantly higher than that of ODN2006. Mechanistic analysis revealed that S-540956 enhanced the induction of MHC class I peptide-specific CD8^+^ T cell responses *via* TLR9 in a CD4^+^ T cell-independent manner. In mice, the therapeutic effect of S-540956-adjuvanted with a human papillomavirus (HPV)-E7 peptide vaccine against HPV-E7-expressing TC-1 tumors was significantly better than that of an ODN2006-adjuvanted vaccine. Our findings demonstrate a novel adjuvant discovery with the complementary strand conjugated to a lipid, which enabled draining LN targeting and increased ODN2006 accumulation in draining LNs, thereby enhancing the adjuvant effect. Our findings imply that S-540956 is a promising adjuvant for cancer peptide vaccines and has a high potential for applications in various vaccines, including recombinant protein vaccines.

## Introduction

Despite the large number of clinical trials of cancer peptide vaccines, no human cancer peptide vaccine has been approved to date (1). To eliminate tumor cells, robust induction of prime and recall T cell-mediated immune responses is essential for cancer peptide vaccines, and secondary lymphoid organs are crucial for orchestrating immune responses (2). Typical components of cancer peptide vaccines include a major histocompatibility complex (MHC)-I-restricted peptide(s) and an adjuvant(s), where the adjuvants act as key players for enhancing CD8^+^ T cell responses. Previous reports indicated that efficient delivery of adjuvants to draining lymph nodes (LNs) increased antigen-presenting cell (APC) activation and robust T cell responses, without increasing systemic toxicity (3, 4).

CpG oligodeoxynucleotides (ODNs) are synthetic single-stranded DNA fragments containing unmethylated CpG motifs that mimic bacterial DNA (5, 6). CpG ODNs can strongly activate plasmacytoid dendritic cells (pDCs) and B cells *via* the Toll-like receptor 9 (TLR9)-signaling pathway and promote the establishment of adaptive immunity (7). The efficacy and tolerability of CpG ODNs have been demonstrated in a large number of clinical trials (8); therefore, CpG ODN modification is a promising approach for developing effective and safe cancer peptide vaccines. Interestingly, a CpG ODN combined with an amphiphilic tail has been reported to enhance the accumulation of draining LNs *via* albumin-hitchhiking and increase CD8^+^ T cell responses, which can maximize the anti-tumor effects of cancer peptide vaccines (3). In fact, the enhanced adjuvant effect provided by combining CpG ODN with amphiphilic tails has also been demonstrated when mixed with a severe acute respiratory syndrome coronavirus 2 (SARS-CoV-2) spike-2 receptor binding domain protein (9). Albumin is intrinsically transferred to draining LNs, and thus, molecules that bind albumin are effectively delivered to draining LNs (10). This albumin-hitchhiking strategy prompted us to further develop and characterize CpG ODNs capable of targeting draining LNs similar to a previous study that directly conjugated cholesterol, or a lipid, to the 5′ end of a CpG ODN and added an ODN to the 5′ end (3). Similarly, a mouse-specific CpG ODN has been evaluated in murine tumor model, however a similar human TLR9-selective CpG ODN has not been previously investigated for cancer peptide vaccines in mice. In addition, the immunological mechanisms have not been fully investigated to identify key players in the adjuvanticity of draining LN-targeting CpG ODNs. However, a previous structure–activity relationship study demonstrated that the agonistic activities of CpG ODNs for TLR9 were altered by modifying the 20-mer oligonucleotide sequence (11). Hence, the direct modification can potentially alter the immunostimulatory effects of CpG ODNs and may cause an unpredictable immunotoxicity.

Therefore, to develop a novel draining LN-targeting adjuvant, we synthetized modified CpG ODNs using different chemical approaches, based on a previous report (3). Specifically, we developed S-540956, which was synthesized by annealing a single complementary DNA strand with an amphiphilic chain unit to ODN2006 without modifying ODN2006 itself (12, 13). We then investigated the delivery and immunological characteristics of S-540956 as a potential cancer peptide vaccine adjuvant.

## Materials And Methods

### Compounds

S-540956, a compound composed of a double-stranded oligodeoxynucleotide, was synthesized by solid-phase synthesis using a typical phosphoramidite method, as described previously (14). Briefly, CpG ODN2006 (5′-TCGTCGTTTTGTCGTTTTGTCGTT-3′) and complementary strands were synthesized using an automated nucleic acid synthesizer (ns-8-II; Ajinomoto-Biopharma Services, Osaka, Japan); lipid ligands were attached to the complementary strand. The complementary strand with the lipid ligands was named C-540956. The nucleotides of ODN2006 and C-540956 were linked *via* phosphorothioate and phosphodiester bonds, respectively. Individually purified ODN2006 and C-540956 were hybridized after briefly heating them to 65°C and subsequently cooling to 20-27°C. The purity of the hybridized oligonucleotides was analyzed using liquid chromatography-mass spectrometry. CpG1018 (B-type CpG) and ODN1826 (B-type CpG specific for murine TLR9) were also synthesized by the automated nucleic acid synthesizer (ns-8-II; Ajinomoto-Biopharma Services). ODN1826 was annealed with the complementary strand attached to the lipid ligands: these were synthesized using the same methods as that for S-540956. Alexa Fluor 647-labeled ODN2006 (ODN2006 AF647) was synthesized by Ajinomoto-Biopharma Services. Alexa Fluor 647-labeled S-540956 (S-540956 AF647) was composed of ODN2006 AF647 hybridized with C-540956. Polyinosinic-polycytidylic acid (polyI:C) was purchased from *In vivo*Gen (Toulouse, France).

### Animals

Six-to eight-week-old female C57BL/6J mice were purchased from CLEA Japan, Inc. (Tokyo, Japan). *Tlr9^-/-^* mice were purchased from Oriental BioService, Inc. (Kyoto, Japan). *Tap1^-/-^* and *Cd4^-/-^* mice were obtained from The Jackson Laboratory (Bar Harbor, ME). All animal studies were conducted following appropriate guidelines and with the approval of the National Institutes of Biomedical Innovation, Health, and Nutrition, as well as the Shionogi Animal Care and Use Committee (Osaka, Japan).

### TLR9 Reporter-Gene Assay

Secreted embryonic alkaline phosphatase (SEAP) reporter HEK-Blue™-hTLR9 cells (expressing human TLR9 and NF-κB-inducible SEAP) and HEK-Blue™-Null1 cells (expressing only NF-κB-inducible SEAP) were purchased from *In vivo*Gen. After activation by treatment with different compounds, secreted SEAP levels were measured using QUANTI-Blue medium (*In vivo*Gen), and the absorbance was measured at 540 nm using a Varioskan Flash multimode reader (Thermo Fisher Scientific, Waltham, MA).

### Surface Plasmon Resonance Analysis

Interactions between S-540956 and human or mouse serum albumin (HSA or MSA, respectively) were analyzed using a BIACORE S51 system (GE Healthcare UK Ltd., Buckinghamshire, UK), according to a previously reported method (15). The sensorgrams for the S-540956 interaction with HSA or MSA were analyzed by curve fitting using numerical-integration analysis. The data were fitted globally by simultaneously fitting the S-540956 sensorgrams obtained at six different concentrations using BI evaluation software (version 4.1). The equilibrium dissociation constant (*K*_D_) values were evaluated by applying linear or nonlinear fitting algorithms to the binding data using the 1:1 Langmuir binding model.

### *In Vivo* Imaging System (IVIS)-Imaging Analysis

Mice were subcutaneously or intramuscularly injected with 5 nmol of S-540956 AF647 or ODN2006 AF647. After injection, the animals were sacrificed, and the draining LNs and spleens were collected at each sampling time. Fluorescence in the collected organs was analyzed using the IVIS imaging system (Perkin Elmer, Waltham, MA).

### Immunofluorescence (IF) Imaging

Mice were intramuscularly injected with 5 nmol of S-540956 AF647, and then the draining LNs were excised after 24 h. The draining LNs were frozen in optimum cutting temperature compound (Sakura Finetek Japan Co., Ltd., Tokyo, Japan). Embedded 8 μm cryostat sections were fixed in cold acetone for 5 min. Anti-CD3 (clone SP7; Nichirei Corporation, Tokyo, Japan), anti-B220 (clone RA3-6B2; BioLegend, San Diego, CA), anti-CD169 (clone 3D6.112; BioLegend), and anti-plasmacytoid dendritic cell antigen-1 ([PDCA-1], JF05 1C2.4.1; Miltenyi Biotech Bergisch Gladbach, Germany) antibodies were used as primary antibodies. AF488-conjugated goat anti-rabbit IgG H&L (Abcam, Cambridge, UK) was used to detect the anti-CD3 antibody, and AF488-conjugated goat anti-rat IgG (minimal cross-reactivity; BioLegend) was used to detect the anti-B220, anti-CD169, and anti-PDCA1 antibodies. After staining the cells, the tissue sections were analyzed using a fluorescence microscope with 10 × and 60 × lenses (BX51; Olympus, Tokyo, Japan). Imaging data were processed using Adobe Photoshop CS2 (Adobe Systems Inc., San Jose, CA).

### Imaging-Stream Analysis

To detect macrophages and pDCs, fluoresceine isothiocyanate (FITC)-conjugated anti-CD11b (cloneM1/70), and phycoerythrin (PE)-conjugated anti-Siglec-H (clone551) were purchased from BioLegend. Imaging-stream analysis was performed as previously described (16). Briefly, mice were intramuscularly injected with 5 nmol of S-540956 AF647 or ODN2006 AF647. Cells collected from draining LNs were stained with FITC anti-CD11b and PE anti-Siglec-H for 30 min at room temperature. Imaging data were obtained using Amnis™ ImageStreamX (Luminex, Austin, TX) and analyzed using IDEAS software (version 6.2; Luminex).

### Splenomegaly Analysis

Mice were intramuscularly injected with 1, 2, or 4 nmol of S-540956 or ODN2006 on day 0, 2, and 4. Three days after the third injection, the mice were sacrificed, and the spleens were collected. The weight of each spleen was measured using an electronic balance.

### Cytokine Measurements

Mice were intramuscularly injected with 5 nmol of S-540956 or ODN2006. Plasma samples were collected 3 and 24 h after injection. The plasma concentrations of TNF-α, IL-6, IFN-γ, and IP-10 were measured using a MILLIPLEX MAP Mouse Cytokine/Chemokine Magnetic Bead Panel-Immunology Multiplex Assay (Merck Millipore, Billerica, MA).

### Immunization and Subsequent Evaluation of Cellular and Humoral Immune Responses

A TRP2_180-188_ peptide (SVYDFFVWL-NH_2_), an OVA_257-264_ peptide (SIINFEKL-NH_2_), and an HPV16-E7_49-57_ peptide (RAHYNIVTF-NH_2_) were synthesized by Sigma-Aldrich Japan K.K. (Tokyo, Japan). Montanide ISA-51 is an incomplete Freund’s adjuvant (IFA) that was purchased from Seppic, Inc. (Fairfield, NJ). OVA protein with low endotoxin level was purchased from FUJIFILM Wako Pure Chemical Co., Ltd. (Osaka, Japan). To evaluate peptide-specific CD8^+^ T cell responses, mice were subcutaneously immunized with 100 µg of TRP2, OVA, or HPV16-E7 peptide mixed with 5 nmol of S-540956 or ODN2006, or 50 µL of Montanide ISA51 on days 0 and 7. Peripheral blood mononuclear cells (PBMCs) were collected 14 days after the first immunization. To evaluate OVA protein-specific CD8^+^ T cell and antibody responses, mice were subcutaneously immunized with 10 μg of OVA protein mixed with 5 nmol of S-540956 or ODN2006 on days 0 and 14. The PBMCs and plasma were collected 21 and 28 days after the first immunization, respectively.

### Flow Cytometric Analysis

FITC-conjugated anti-CD86 (clone GL1, BD Pharmingen, San Diego, CA), brilliant violet (BV)-421-conjugated anti-F4/80 (clone T45-2342 BD Pharmingen), PE-conjugated anti-Siglec-H (clone 551 BL), and BV-605-conjugated anti-CD11b (clone M1/70, BioLegend) antibodies were used to detect CD86 expression in pDCs and macrophages. To analyze cytokine-expressing cells, allophycocyanin–cyanine (Cy)7-conjugated anti-CD-3ϵ (clone 2C11), BV-510-conjugated anti-CD4 (clone RM4-5), BV-570-conjugated anti-CD8α (clone 53-6.7), PE-conjugated anti-IL-2 (clone JES6-5H4), and PE-Cy7-conjugated anti-TNFα (clone MP6-XT22) antibodies were purchased from BioLegend, and an FITC-conjugated anti-IFN-γ (clone XMG1.2) antibody was purchased from BD Pharmingen. For peptide-specific T cell receptor detection, TRP2_180-188_ peptide, OVA_257-264_ peptide, or HPV16-E7_49-57_ peptide loaded PE-labeled tetramers were purchased from Medical & Biological Laboratories, Co. Ltd. (Nagoya, Japan). To detect CD107a expression, BV786-conjugated anti-CD107a antibody was used (clone 1D4B, BD Biosciences, Franklin Lakes, NJ). For tetramer staining, the collected PBMCs were incubated with PE-labeled tetramers and antibodies for 30 min at room temperature. For intracellular cytokine-staining assays, the collected PBMCs were stimulated with cognate peptides for 6 h; next, Golgi-Plug and Golgi-Stop were added, and the PBMCs were incubated for 30 min at room temperature. The cells were fixed and permeabilized using a Cytofix/Cytoperm Kit (BD Biosciences). Dead cells were excluded by LIVE/DEAD™ Fixable Aqua Dead Cell Stain Kit (Thermo Fisher). Data were collected using an LSRII flow cytometer (BD Biosciences) and analyzed using FlowJo software (version 9.8.2; Tree Star, Ashland, OR).

### Measurement of Antibody Titers

Total plasma anti-OVA IgG, IgG1, and IgG2c titers were determined by performing enzyme-linked immunosorbent assays (ELISAs) as described previously (17), with the following modifications. Briefly, 384-well plates were coated with 10 μg/mL OVA antigen solution (FUJIFILM Wako Pure Chemical Co., Ltd, Osaka, Japan) overnight at 4°C. The plates were washed and incubated for 1 h with blocking buffer (phosphate-buffered saline [PBS] containing 1% bovine serum albumin). After blocking, the plates were washed and incubated with 5-fold serially diluted plasma for 2 h. To detect the bound antibodies, the plates were washed and incubated for 1 h with horseradish peroxidase-conjugated anti-mouse total IgG, IgG1, or IgG2c Ab (Bethyl Laboratories, Inc., Montgomery, TX). After the plates were washed, 1-Step Ultra TMB-ELISA solution (Thermo Fisher) was added to each well to initiate the color reaction. The reaction was stopped after 5 min by the addition of 1 M sulfuric acid, and the optical density at 450 nm (OD_450_) was measured using SpectraMax^®^ iD3 device (Molecular Devices, LLC, San Jose, CA). The titer was defined as the highest dilution factor with an OD value of > 0.1.

### Depletion of pDCs, CD4^+^ Cells, CD8^+^ Cells, and Macrophages

An intraperitoneal injection with 500 µg of anti-PDCA1 (clone 927, BioLegend) or intravenous injection with 100 µg of anti-CD4 (clone GK1.5, BioLegend) was performed to deplete pDCs or CD4^+^ cells 1 day before immunization with the vaccine. To deplete CD8^+^ cells, an intraperitoneal injection with 100 µg of anti-CD8α (clone 2.43, Bio X cell, Lebanon, NH) was performed at 15, 17, 19, and 21 days after the inoculation of tumor. To deplete macrophages, 200 µL of clophosome-A (FormuMax Scientific, Inc., Sunnyvale, CA) was intravenously administered on days 1 and 6 before immunization with the vaccine.

### Tumor Model

B16F10 melanoma cells expressing TRP2 (2 × 10^5^ cells) or TC-1 tumorigenic cells expressing HPV16-E6 and E7 (3 × 10^5^ cells) were subcutaneously inoculated into the flanks of mice. Tumor-bearing mice were subcutaneously immunized twice with 100 µg of TRP2_180-188_ or HPV16-E7_49-57_ peptide mixed with 5 nmol of S-540956 or ODN2006. Tumor sizes were measured using an electronic scale and calculated using the following formula: tumor size = tumor length × tumor width^2/2. Two-tailed Student’s *t*-test was used for two-group comparisons. For groups of three or more, one-way analysis of variance (ANOVA) followed by Tukey’s test was used. Statistical significance was set at a P value of < 0.05. Statistical analysis was performed using GraphPad Prism 9 (San Diego, CA).

## Results

### The Complementary Strand With an Amphiphilic Chain Unit Increases ODN2006 Accumulation in the Draining LNs

**Figure 1:**
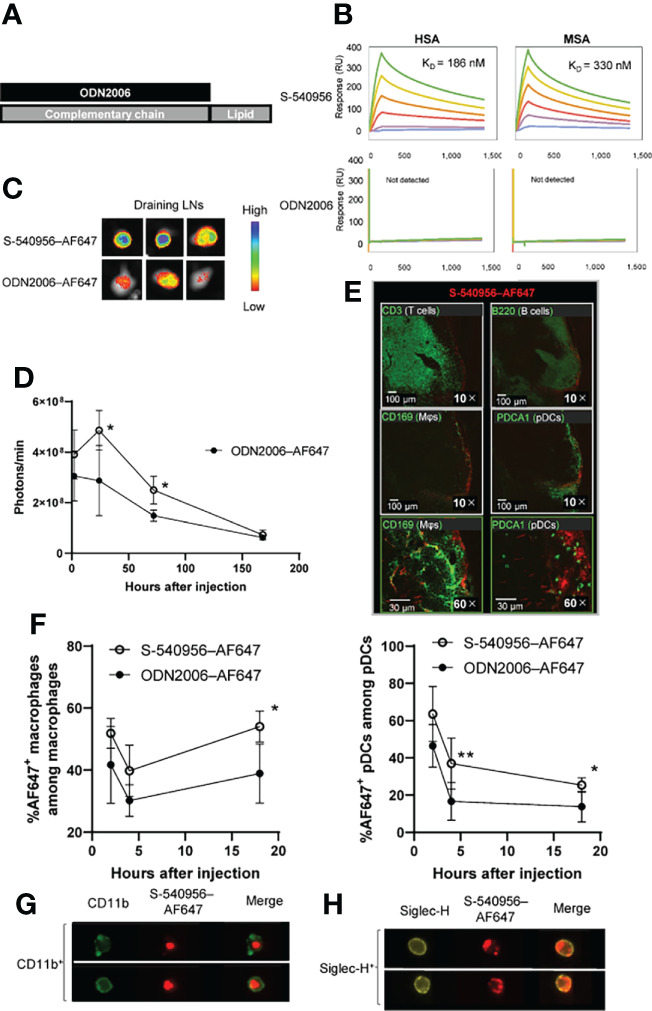
Delivery of S-540956–AF647 to the draining LNs. **(A)** Schematic representation of S-540956. **(B)** Measurements of S-540956- and ODN2006- binding to immobilized HSA and MSA using the BIACORE S51 system. K_D_ values were calculated. **(C, D)** Mice were intramuscularly injected with S-540956–AF647 or ODN2006–AF647. Draining LNs were collected and analyzed at 2, 24, 72, and 168 h after injection (N = 3–5). The images shown represent draining LNs at 24 h after the intramuscular injection **(C)**. The fluorescence intensities of the draining LNs were measured using the IVIS spectrum system **(D)**. **(E)** T cells (CD3^+^), B cells (B220^+^), macrophages (CD169^+^), and pDCs (PDCA^+^) in the draining LNs were analyzed by immunofluorescence imaging at 24 h after the intramuscular injection of S-540956–AF647. **(F)** Incorporation of S-540956–AF647 or ODN2006–AF647 by CD11b^+^ F4/80^+^cells (macrophages) or Siglec-H^+^ CD11c^+^ cells (pDCs) in the draining LNs was analyzed by flow cytometric analysis at 2, 4, and 18 h after the intramuscular injection (N = 4–6). **(G, H)** Localization of S-540956–AF647 in CD11b^+^ cells or Siglec-H^+^ cells in draining LNs was imaged using ImageStreamX software at 4 h after the intramuscular injection of S-540956–AF647. **(D, F)** The data shown indicate the mean ± standard error (SE). *P < 0.05 or **P < 0.01, versus ODN2006–AF647; Student’s *t*-test. Data are representative of two independent experiments.

Direct binding of the amphiphilic chain to CpG ODNs has been reported to enhance its accumulation in draining LNs by binding to albumin after injection, leading to APC activation and increased T cell responses in draining LNs (3). In this study, we designed an amphiphilic chain bound to the complementary strand of ODN2006 (B-type CpG ODN), and its efficacy and safety profiles have been confirmed in clinical trials (8). The ODN2006-annealed complementary strand with an amphiphilic chain unit was named S-540956 (**Figure 1A**). The *in vitro* effect of S-540956 on the activity of the TLR9 signaling pathway was compared with that of ODN2006 using HEK-Blue™ hTLR9 cells, which stably express the TLR9 gene. The half-maximal effective concentration values of S-540596 and ODN2006 were 300 and 180 nM, respectively (**Figure S1A**). C-540956, comprised of a complementary strand with an amphiphilic chain unit, did not activate the TLR9 signaling pathway (data not shown). TLR9-independent stimulation was not observed in HEK-Blue™-Null1 cells after incubation with S-540956, ODN2006, or C-540956 (**Figure S1B**). The mean at OD 620 nm of the positive control (tumor necrosis factor-α [TNF-α]) and negative control (buffer) samples were 2.60 and 0.11, respectively. These results suggest that the hybridization of the complementary strand with the amphiphilic chain to ODN2006 did not alter the *in vitro* effect of ODN2006 on TLR9 signaling. Interactions between S-540956 and HSA or MSA were analyzed by performing surface plasmon resonance (SPR) assays. The K_D_ values for the binding of S-540956 to HSA and MSA were 186 nM and 330 nM, respectively (**Figure 1B**). In contrast, a measurable K_D_ value could not be determined for the interactions between ODN2006 and HSA or MSA. The data from the SPR assays supported the concept of a delivery system for S-540956, based on the kinetics of albumin for efficient delivery to the draining LNs. To investigate the draining LN-targeting profile of S-540956, we examined S-540956 accumulation in the draining LNs using the IVIS imaging system. AF647-conjugated S-540956 or AF647-conjugated ODN2006 was injected *via* intramuscular, subcutaneous, or intravenous routes. S-540956–AF647 accumulated in the draining LNs at statistically higher levels than did ODN2006–AF647 following an intramuscular or subcutaneous injection, from 24 to 48 h post-injection (*P* < 0.05, **Figures 1C, D**). AF647 fluorescence was not detected in the draining LNs after intravenous injection of S-540956–AF647 or ODN2006–AF647 (data not shown). To examine the systemic distribution of S-540956, the accumulation of S-540956–AF647 in the spleen was also assessed after an intramuscular injection. S-540956–AF647 accumulation in the spleen was significantly lower than that of ODN2006–AF647 (*P* < 0.01, **Figures S2A, B**). The systemic distribution after a subcutaneous injection was consistent with that following an intramuscular injection (**Figures S2C, D**). A low systemic distribution profile is associated with reduced systemic toxicity (3). To assess the systemic toxicity of S-540956, splenomegaly was investigated after three repeated injections of S-540956 at 0, 2, and 4 days post-injection. The spleen weights of S-540956-injected mice were significantly lower than that of ODN2006-injected mice at a dose of 4 nmol (*P* < 0.01, **Figures S2E, F**). Systemic proinflammatory cytokine responses were measured in plasma samples after the intramuscular injection. The concentrations of TNF-α and IP-10 in S-54056-injected mice were significantly lower than those in ODN2006-injected mice at 3 h after injection (*P* < 0.05, **Figures S3A, D**). No statistical differences between S-540956- and ODN2006-injected mice were observed in terms of the TNF-α, IL-6, IFN-γ, and IP-10 concentrations at 24 h after injection (**Figures S3A–D**). The splenomegaly and cytokine-response data suggest that the draining LN-targeting profile of S-540956 did not increase the systemic toxicity of ODN2006 in mice.

TLR9 is expressed in DCs, macrophages, and B cells in mice, and these APCs orchestrate innate immune responses and contribute to the establishment of adaptive immunity (5, 7). Next, we focused on cells that incorporated S-540956 in the draining LNs. The distribution of S-540956 in the draining LNs was analyzed by IF imaging focusing on B cells (B220^+^), macrophages (CD169^+^), pDCs (Siglec-H^+^), and T cells (CD3^+^). The majority of S-540956–AF647 signal was detected on the surface of draining LNs and distributed to the B cell zone, localized to macrophages and pDCs (**Figure 1E**). We next examined the incorporation of S-540956–AF647 by macrophages or pDCs in the draining LNs by flow cytometry and ImageStreamX software. S-540956–AF647 was incorporated by pDCs and macrophages at higher levels, when compared to ODN2006 (**Figure 1F** and **Figure S4**), and S-540956–AF647 was detected in pDCs and macrophages, as determined by ImageStreamX software (**Figures 1G, H**). Taken together, the intrinsic property of albumin to translocate to the draining LNs (10) and our findings of the albumin binding and the enhanced accumulation of ODN2006 in the draining LNs by annealing the complementary strand with the amphiphilic chain unit suggest that S-540956 (injected into the muscle or subcutaneous tissue) translocated to the lymph vessels after binding albumin at the injection site and efficiently accumulated in the draining LNs.

### The Draining LN-Targeting Profile Shows That ODN2006 Enhances pDC Activation in Draining LNs

**Figure 2:**
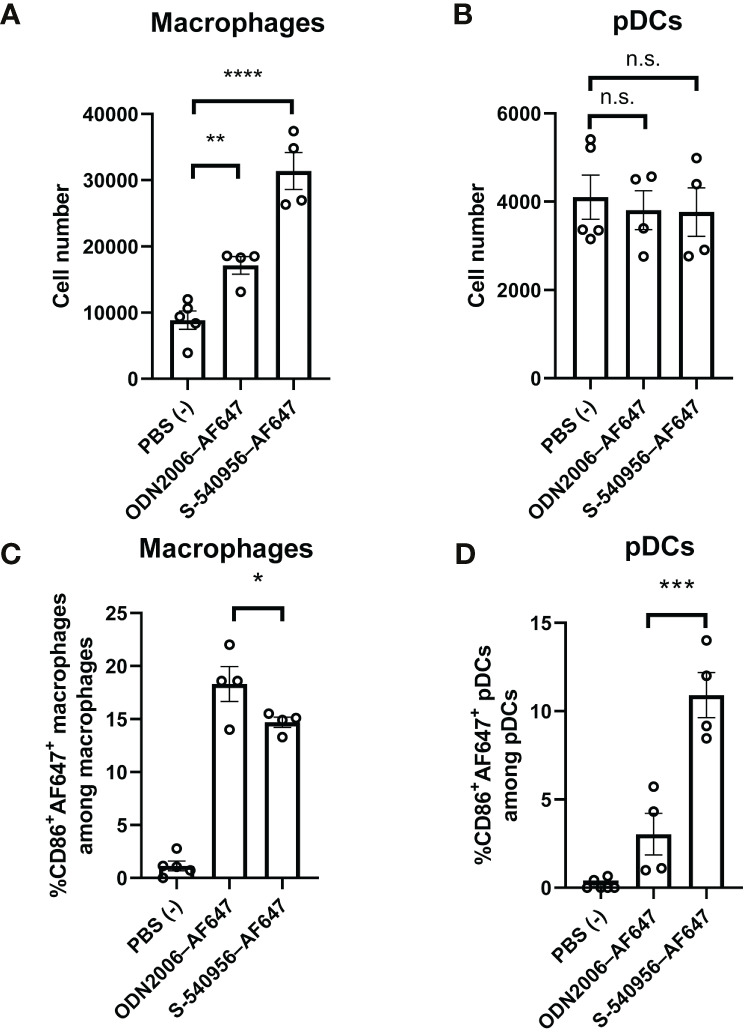
S-540956 activated APCs in draining LNs. Mice were intramuscularly injected with S-540956–AF647 or ODN2006–AF647, and then draining LNs were collected at 18 h after an injection (N = 4–6). The numbers of **(A)** macrophages and **(B)** pDCs and **(C)** CD86^+^ AF647^+^ macrophages (F4/80^+^, CD11b^+^) or **(D)** CD86^+^ AF647^+^ pDCs (CD11c^+^, Siglec-H^+^) in the draining LNs were measured by flow cytometry. The data shown indicate the mean ± SE. *P < 0.05, **P < 0.01, ***P < 0.005, ****P < 0.001; one-way ANOVA. Data are representative of two independent experiments. not statistically significant (n.s.).

Imaging analysis revealed that S-540956 was incorporated into macrophages and pDCs after an injection. Next, we focused on macrophages (F4/80^+^, CD11b^+^) and pDCs (CD11c^+^, Siglec-H^+^) in the draining LNs to further dissect the differences between S-540596 and ODN2006 in terms of their abilities to activate APCs. CD86 expression in both macrophages and pDCs was measured by flow cytometry to evaluate the effects of S-540956 on their maturation. The results showed that the number of macrophages in S-540956 injected mice was higher than that in ODN2006 at 18 h after injection (**Figure 2A**). The number of pDCs was not increased after injection of S-540956 (**Figure 2B**). S-540956 enhanced CD86 expression in both macrophages and pDCs (**Figures 2C, D**, **Figure S4**) and that CD86 expression in pDCs from S-540956-injected mice was significantly higher than that in ODN2006-injected mice (*P* < 0.01, **Figure 2D**). These data suggest that S-540956 strongly promoted pDC activation (rather than macrophage activation) in draining LNs.

### The Draining LN-Targeting Profile Reveals That ODN2006 Enhances the Induction of CD8^+^ T Cell Responses to MHC Class I-Restricted Cancer Peptides

**Figure 3:**
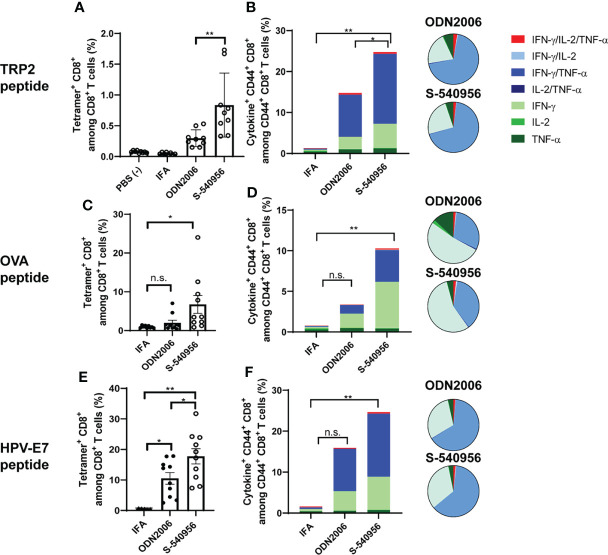
S-540956 enhanced MHC class-I-restricted cancer peptide-specific CD8^+^ T cell responses. Mice were intramuscularly immunized with peptide/S-540956, ODN2006, or Montanide ISA-51 on days 0 and 7 (N = 8–9). **(A, C, E)** TRP2, OVA, or HPV-E7 peptide-specific CD8^+^ T cells among PBMCs and **(B, D, F)** polyfunctional CD8^+^ cells in splenocytes were analyzed by flow cytometry on day 14. **(A, C, E)** The data shown indicate the mean ± SE. **(B, D, F)** The bar graphs indicate the mean, and the pie charts show the ratios of different cytokines expressed by CD44^+^ CD8^+^ cells. n.s., not statistically significant, *P < 0.05, **P < 0.01, as determined by one-way ANOVA. Data are representative of two independent experiments.

The complementary strand with an amphiphilic chain unit enhanced ODN2006 accumulation in the draining LNs and pDC activation. Activated pDCs carrying antigens can promote robust priming and T cell differentiation in LNs (18). This evidence prompted us to evaluate S-540956 as a vaccine adjuvant. Both humoral and cellular responses were investigated after immunization with a recombinant OVA antigen or peptide, mixed with S-540956. Both OVA-specific antibody levels (**Figures S5A–C**) and CD8^+^ T cell responses (**Figure S5D**) were elevated after the addition of S-540956, and the adjuvanticity of S-540956 was statistically higher than that of ODN2006 (**Figures S5A, C, D**; *P* < 0.01, **Figure S5B**; *P* < 0.05). The adjuvant effect of S-540956 was also compared with that of CpG1018, which is contained in HEPLISAV-B launched as a hepatitis B vaccine (19). The OVA-specific CD8^+^ T cell response enhanced by S-540956 was statistically higher than that by CpG1018 (**Figure S5E**; *P* < 0.01). Consistent with the results obtained with the recombinant OVA protein, S-540956 adjuvant enhanced the induction of CD8^+^ T cell responses to TRP2 peptide (**Figure S6**, *P* < 0.001), and the adjuvanticity of S-540956 was statistically higher than that of ODN2006 (*P* < 0.05). Each TRP2, OVA, or HPV-E7 peptide-specific CD8^+^ T cell responses induced by S-540956 was higher than that by IFA or ODN2006 (**Figures 3A, C, E**). To evaluate the versatility of our chemical approach, we synthesized ODN1826 annealed with the complementary strand with an amphiphilic chain unit (ODN1826-C1826). TRP2 peptide-specific CD8^+^ T cell responses were measured after immunization with ODN1826-C1826-adjuvanted vaccine. The complementary strand with an amphiphilic chain unit enhanced the adjuvanticity of ODN1826 to induce TRP2 peptide-specific CD8^+^ T cell responses (**Figure S7**). Polyfunctional effector CD8^+^ T cells are thought to secrete multiple cytokines and cytotoxic markers and are associated with effective anti-tumor effects in humans (20, 21). To evaluate the quality of the CD8^+^ T cells, the polyfunctionality of CD8^+^ T cells was also investigated. The ratios of double- (IFN-γ/IL-2, IFN-γ/TNF-α, and IL-2/TNF-α) and triple- (IFN-γ/IL-2/TNF-α) positive CD44^+^ CD8^+^ T cells among cytokine-positive CD44^+^ CD8^+^ T cells specific to TRP2, OVA, and HPV-E7 peptides in the S-540956-adjuvanted vaccine groups were higher than those in the IFA-adjuvanted vaccine groups (**Figures 3B, D, F**). In addition, the peptide-specific CD107a^+^ CD8^+^ T cell responses in S-540956-adjuvanted vaccine groups were higher than those of ODN2006-adjuvanted vaccine groups (**Figures S8A–C**). No significant differences were observed in the ratios of double- and triple-positive CD44^+^ CD8^+^ T cells among cytokine-positive CD44^+^ CD8^+^ T cells between mice treated with S-540956 or ODN2006. These data suggest that draining LN targeting enhanced the adjuvant effect of ODN2006 to induce CD8^+^ T cell responses but did not alter the quality of the CD8^+^ T cells.

### S-540956 Enhances CD8^+^ T Cell Responses to the Cancer Peptide Vaccine in a pDC-Dependent Manner

**Figure 4:**
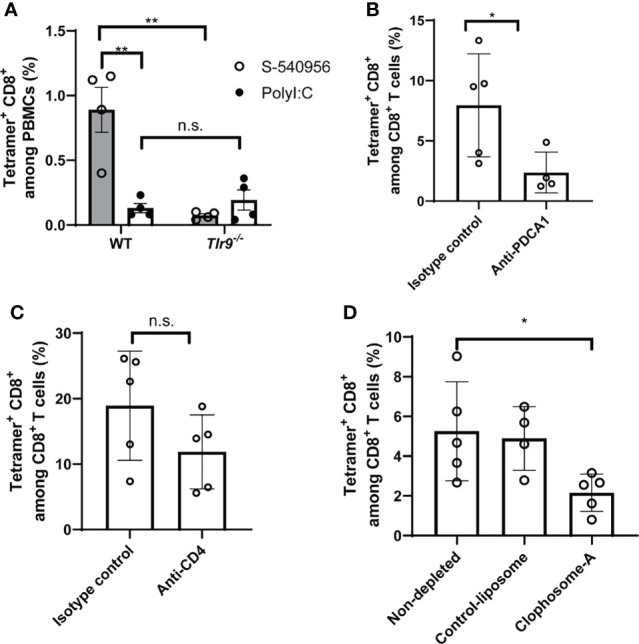
S-540956 enhanced TRP2-specific CD8^+^ T cell responses in TLR9- and pDC-dependent manners. **(A)** Wild-type (WT) or *Tlr9^-/-^* mice were subcutaneously immunized with TRP2 peptide/S-540956 or TRP2 peptide/PolyI:C on days 0 and 7 (N = 4). TRP2 peptide-specific CD8^+^ T cells were measured by flow cytometry using a TRP2 tetramer, on day 14. **(B)** pDCs or **(C)** CD4^+^ T cells were depleted by administering an anti-PDCA1 or anti-CD4 antibody (N = 4–5) to WT mice immunized with TRP2 peptide/S-540956, and TRP2 peptide-specific CD8^+^ T cells were measured as described in **(A)**. **(D)** Macrophages were depleted by administering clophosome-A to WT mice immunized with TRP2 peptide/S-540956, and TRP2 peptide-specific CD8^+^ T cells were measured as described in **(A)**. The data shown indicate the mean ± SE. n.s., not statistically significant, *P < 0.05, **P < 0.01, as determined by Student’s *t*-test or one-way ANOVA. Data are representative of two independent experiments.

Activation of the TLR9-signaling pathway by CpG ODNs can enhance the induction of antigen-specific CD8^+^ T cell responses (22). To provide insights into how S-540956 strongly induced CD8^+^ T cell responses, we next investigated the mechanisms involved in the adjuvanticity of S-540956. *Tlr9^-/-^* mice were immunized with a TRP2 peptide mixed with S-540956. CD8^+^ T cell responses induced by S-540596 decreased in *Tlr9^-/-^* mice, suggesting that the mechanisms that induced CD8^+^ T cell responses were not altered by the addition of complementary strands with amphiphilic chain units and that other signaling pathways were not involved in the induction of CD8^+^ T cell responses (**Figure 4A**). To further understand which cells acted as key players in the adjuvanticity of S-540956, pDCs and CD4^+^ T cells were depleted by injection of anti-PDCA-1 and anti-CD4 antibodies, respectively, and macrophages were depleted using clophosome-A (**Figures 4B–D** and **Figures S9A, B, D**). CD8^+^ T cell responses were reduced by depleting pDCs (**Figure 4B**) or macrophages (**Figure 4D**). Depleting CD4^+^ T cells did not affect CD8^+^ T cell induction by S-540956 (**Figure 4C**). Collectively, these results suggest that the immunological mechanisms whereby S-540956 enhanced the induction of MHC class I-restricted peptide-specific CD8^+^ T cell responses were not altered by the addition of complementary strands with amphiphilic chain units and that CD4^+^ T cells were not required for inducing MHC class I-restricted peptide-specific CD8^+^ T cell responses.

### The Therapeutic Effect of the Cancer Peptide Vaccine Is Significantly Enhanced by S-540956 in a CD4^+^ T Cell-Independent Manner

**Figure 5:**
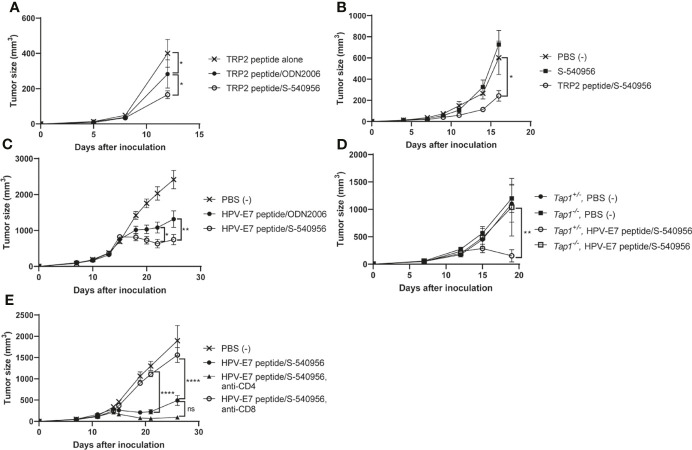
Adjuvant effect of S-540956 with a cancer peptide vaccine against tumors in mice. **(A)** Mice were subcutaneously immunized with TRP2 peptide alone, TRP2 peptide/ODN2006, or TRP2/S-540956 on days 0 and 7 after the first immunization (N = 8). The mice were subcutaneously inoculated with TRP2-expressing B16F10 melanoma cells on day 14. **(B)** Mice were subcutaneously inoculated with TRP2-expressing B16F10 melanoma cells. The mice were subcutaneously immunized with PBS (–), S-540956, or TRP2/S-540956 at 7 and 14 days after inoculation (N = 9–10). **(C)** Mice were subcutaneously inoculated with HPV-E7-expressing TC-1 tumor cells. The mice were intramuscularly immunized with PBS (–) or HPV-E7 peptide mixed with S-540956 or ODN2006 at 9 and 16 days after inoculation (N = 8). **(D)** *Tap1^+/-^* or *Tap^-/-^* mice were subcutaneously inoculated with TC-1 tumor cells. The mice were subcutaneously immunized with PBS (–) or HPV-E7/S-540956 at 7 and 14 days after inoculation (N = 3–5). **(E)** Mice were subcutaneously inoculated with HPV-E7-expressing TC-1 tumor cells. The mice were intramuscularly immunized with PBS (–) or HPV-E7 peptide mixed with S-540956 at 7 and 14 days after inoculation. CD4^+^ cells or CD8^+^ cells were depleted by administrating anti-CD4 antibody or anti-CD8 antibody (N = 6–8). Tumor sizes were measured using a vernier caliper. The data shown indicate the mean ± SE. *P < 0.05, **P < 0.01, ****P < 0.001, as determined by one-way ANOVA. Data are representative of two independent experiments.

Our immunological analysis suggested that S-540956 strongly enhanced the anti-tumor effect of the MHC class I-restricted cancer peptide vaccine, when compared to ODN2006 (**Figures 3A–F**) and polyI:C (**Figure 4A**), which have been previously used as cancer peptide vaccines (8, 23). The anti-tumor effect of the S-540956-adjuvanted peptide vaccine was evaluated in TRP2-expressing B16F10 melanoma cell- or HPV-E7-expressing TC-1 tumor cell-grafted mice. The anti-tumor effect of the TRP2 peptide vaccine was significantly enhanced by the addition of S-540956, and the adjuvanticity of S-54095 was statistically higher than that of ODN2006 (**Figure 5A**, *P* < 0.05). S-540956 alone did not show anti-tumor effect in this model (**Figure 5B**). Consistent with the results in mice administered B16F10 melanoma cells, the anti-tumor effect of the HPV-E7 peptide vaccine was enhanced by S-540956, and the adjuvant effect of S-540956 was statistically higher than that of ODN2006 (**Figure 5C**, *P* < 0.01). The anti-tumor effect of the S-540956-adjuvanted vaccine was significantly reduced in *Tap1^-/-^* mice (**Figure 5D**). In addition, the depletion of CD8^+^ T cells and not CD4^+^ T cells reduced the anti-tumor effect of S-540956-adjuvanted vaccine (**Figures 5E** and **Figures S9C, D**). These results strongly suggest that the anti-tumor effect of the S-540956-adjuvanted vaccine depends on MHC class I peptide-specific CD8^+^ T cells.

## Discussion

Cancer peptide vaccines represent one of the strategies used to control cancers by inducing robust MHC class I-restricted peptide-specific CD8^+^ T cells with long-lasting responses to overcome the tumor-immunosuppressive environment. To eradicate tumor cells with high specificity, peptides are designed by exploring tumor-associated antigens, tumor-specific antigens, and neoantigens important for interactions between tumor cells and immune responses (2). Recently, in addition to monotherapy with cancer peptide vaccines, cancer peptide vaccines have been investigated for combination therapy with immune-checkpoint inhibitors (ICIs) with the objective of improving the clinical outcomes of ICIs (24). Adjuvants are crucial components of cancer peptide vaccines as they promote, prime, and recall peptide-specific CD8^+^ T cell responses (25). To enhance the adjuvanticity, LN-targeting adjuvants have been explored by directly modifying TLR7/8 or TLR9 ligands (3, 26). These chemical modifications may alter the immunostimulatory effects of ligands, however, it is difficult to predict the immunotoxicity that may be attributable to the modified chemical structures in humans, based on pre-clinical research. Therefore, we elected to not modify the ligands and instead developed S-540596 by annealing complementary strands with amphiphilic chain units to ODN2006, one of the most commonly used adjuvants for cancer peptide vaccines in clinical trials (27, 28). Improved immunogenicity was confirmed; however, ODN2006-adjuvanted cancer peptide vaccines did not exhibit significant induction of cytotoxic T lymphocytes (CTLs) in patients, and no ODN2006 adjuvanted cancer peptide vaccine has been approved for clinical use till date. These clinical studies indicate the necessity of improving the adjuvanticity of ODN2006 for developing more effective cancer peptide vaccines. In this study, S-540956 exhibited more potent adjuvanticity than ODN2006 when evaluating the efficacies of cancer peptides against HPV-E7-expressing TC-1 tumors in mice. This promising result suggests that the S-540956-adjuvanted cancer peptide vaccine will show greater efficacy than ODN2006 against tumor cells in clinical settings.

TLR9 can recognize single-stranded DNA (ssDNA) and activate related signaling pathways originating from endosomes (6). Comparable stimulation of TLR9 signaling was observed between the double-stranded DNA (dsDNA) molecule, S-540956, and the ssDNA molecule, ODN2006, in HEK-Blue TLR9 cells. In this study, the nucleotides in ODN2006 are connected by a phosphorothioate linkage based on a previous report (29). The use of phosphorothioate nucleotides enhances the resistance of CpG ODNs to DNases when compared with the phosphodiester bond-linked nucleotides, which are components of native DNA (30). The nucleotides of the complementary strand (C-54056) for S-540956 were joined by phosphodiester linkage. A recent study indicated that DNase II in endosomes digests a phosphodiester linker of CpG ODN, in which the nucleotides at the center were joined by phosphodiester linkages with a phosphorothioated backbone at both ends, and the digested CpG ODN activates TLR9 (31). Hence, C-540956 is considered to be digested by DNase II in the endosomes, and the released ODN2006 stimulated TLR9 signaling pathway.

pDCs and conventional DCs are heterogeneous subsets of DCs that orchestrate innate and adaptive immune responses. pDCs can sense ssDNA *via* TLR9 and have diverse functions, which promote the establishment of optimal cellular immunity by presenting antigens to T cells (32). The delivery of antigen-targeting pDCs in combination with TLR agonists can enhance the induction of CD8^+^ T cell responses (33). Our immunological analysis revealed differences between S-540956 and ODN2006 in terms of adjuvant incorporation by pDCs and activation of pDC, and the adjuvanticity of S-540956 diminished after the pDCs were depleted. A large number of DCs and CD8^+^ T cells are considered to reside in LNs, when compared to those in peripheral tissues, and the transportation of antigens and adjuvants from the injection site to LNs is important for enhancing vaccine efficacy (34, 35). TLR9 is expressed in both mouse and human pDCs (36); therefore, we expect that S-540956 shows potent adjuvanticity in humans. Our immunological analysis indicated that the efficient activation of pDCs in LNs after the high accumulation of S-540956 is a critical innate immune response that enables S-540956 to induce more robust cellular-immune responses than ODN2006.

Some adjuvants can induce excessive proinflammatory cytokine responses related to cytokine storms and systemic inflammation (37). Excess systemic immune responses induced by frequent injections of CpG ODN cause splenomegaly, which is mainly due to erythroid and myeloid expansion in the red pulp, and consequently lead to extramedullary hematopoiesis (38, 39). Although proinflammatory cytokine responses were observed in the blood after the S-540956 injection, each induction level was not statistically higher than that of ODN2006. In addition, splenomegaly induced by frequent injections of S-540956 was lower than that induced by ODN2006. ODN2006 is a well-known safe adjuvant that has been use in a large number of clinical trials (8). For instance, ODN2006 has been used as a vaccine adjuvant for the recombinant AMA1-C1/alhydrogel malaria vaccine. This addition of ODN2006 was found to significantly enhance the humoral immune responses; however, local and systemic adverse events were also increased (40). It is, therefore, necessary to reduce the number of adverse events induced by the adjuvant, particularly for preventive vaccines. The targeting of LNs by S-540956 might decrease the number of adverse events and thereby, regulate the severity of adverse event, especially systemic adverse effects. Therefore, we expect that S-540956 poses a low risk for inducing systemic inflammation and splenomegaly in humans.

Collectively, our data supports the conclusion that the targeting LN profile of S-540956 can elicit potent adjuvanticity without inducing excess systemic inflammation and immunotoxicity. However, future investigations into the safety profile of S-540956 *via* clinical trials are expected to further confirm the benefit of an LN-targeting profile as a vaccine adjuvant.

In conclusion, the results of this study demonstrated a chemical approach for discovering a novel adjuvant in which the complementary strand was conjugated to a lipid for draining LN targeting, which increased ODN2006 accumulation in the draining LNs and consequently enhanced the adjuvant effect, without elevating systemic inflammation.

## Data Availability Statement

The original contributions presented in the study are included in the article/**Supplementary Material**. Further inquiries can be directed to the corresponding author.

## Ethics Statement

The animal study was reviewed and approved by National Institutes of Biomedical Innovation, Health, and Nutrition (NIBIOHN) and Shionogi Animal Care and Use Committee.

## Author Contributions

TN, TT, ST, TK, and YI designed and conducted the experiments, performed the data analysis, interpreted the results, and wrote the manuscript. TN and MO performed the data analysis, interpreted the results, and wrote the manuscript. TN, TI, AK, KK, TY, MN, and KI conceived the project, designed the experiments, interpreted the results, and revised the manuscript. All authors contributed to the manuscript and approved the submitted version.

## Funding

This work was supported by Shionogi & Co., Ltd., and in part by NIBIOHN, Core Research for Evolutional Science and Technology (CREST)/Japan Science and Technology Agency (JST), and Japan Agency for Medical Research and Development (AMED).

## Conflict of Interest

TN, TT, MO, ST, TK, YI, TI, AK, KK, and MN are employees of Shionogi & Co., Ltd.

The remaining authors declare that the research was conducted in the absence of any commercial or financial relationships that could be construed as a potential conflict of interest.

The authors declare that this study received funding from Shionogi & Co., Ltd. The funder had the following involvement in the study: study design, collection, analysis, interpretation of data, the writing of this article or the decision to submit it for publication.

## Publisher’s Note

All claims expressed in this article are solely those of the authors and do not necessarily represent those of their affiliated organizations, or those of the publisher, the editors and the reviewers. Any product that may be evaluated in this article, or claim that may be made by its manufacturer, is not guaranteed or endorsed by the publisher.
