# Supplementary material for: S-540956, a CpG Oligonucleotide Annealed to a Complementary Strand With an Amphiphilic Chain Unit, Acts as a Potent Cancer Vaccine Adjuvant by Targeting Draining Lymph Nodes
Source: Front Immunol. 2021 Dec 23;12:803090. doi: 10.3389/fimmu.2021.803090 (PMC8735836; doi:10.3389/fimmu.2021.803090)
Supplement: Supplementary file 1 [file DataSheet_1.pdf]

Supplementary Material

1 Supplementary Figures

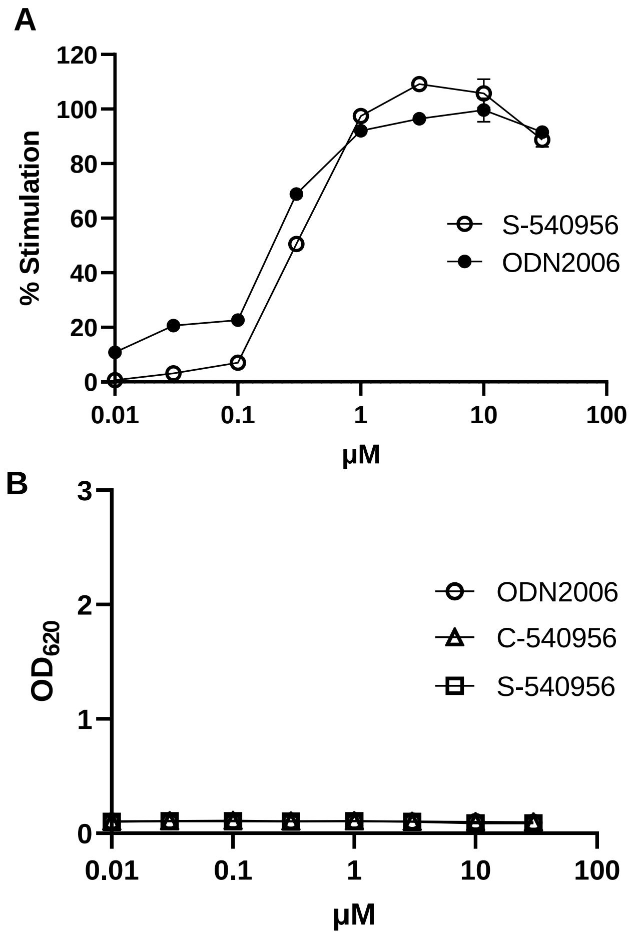

Supplementary Figure 1. *In vitro* effect of S-540956 on TLR9 signaling
(A) HEK-Blue™ hTLR9 cells stably expressing the TLR9 gene and (B) HEK-Blue™ Null1 cells were stimulated with S-540956, ODN20006, or C-540956 for 16–20 h (N = 2). The data shown indicate the mean ± standard deviation (SD). Secreted embryonic alkaline phosphatase (SEAP) production was measured by measuring OD 620 nm values. Data are representative of two independent experiments.

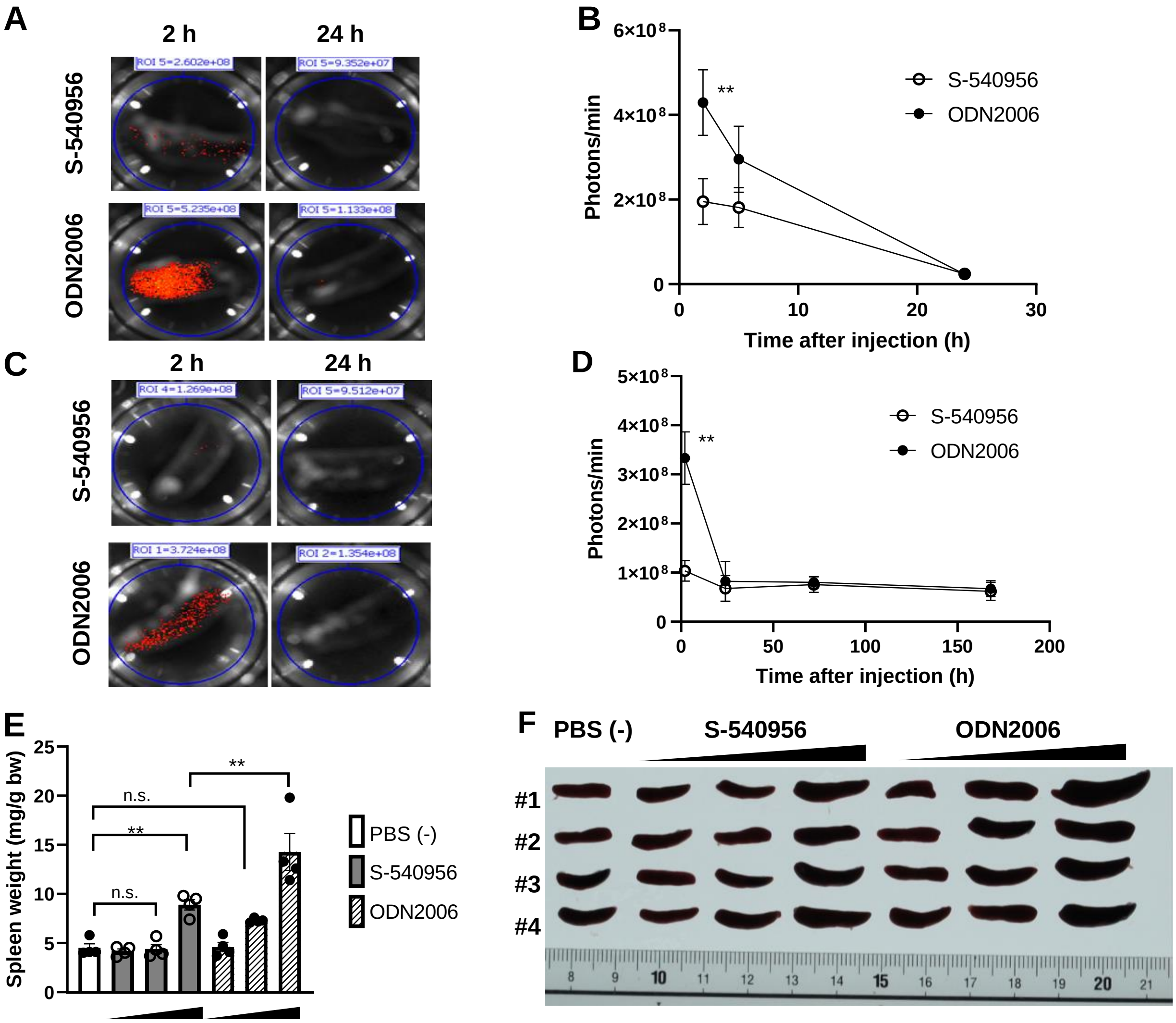

Supplementary Figure 2. Systemic distribution and toxicity after S-540956 injection
(A–D) Spleens were collected and analyzed at 2, 6, and 24 h after intramuscular injection. The fluorescence intensities of the spleens were calculated using an IVIS spectrum system. Panels A and B show data obtained after a subcutaneous injection. Panels C and D show data obtained after an intramuscular injection (N = 3–6). (E, F) Mice were intramuscularly injected with 1, 2, or 4 nmol of S-540956 or ODN2006 at 0, 2, and 4 days after the first injection (N = 4). At 7 days after the first injection, the spleens were excised and weighed. The data shown indicate the mean ± standard error (SE). n.s., not statistically significant, $**P < 0.01$, as determined by one-way analysis of variance (ANOVA). Data are representative of two independent experiments.

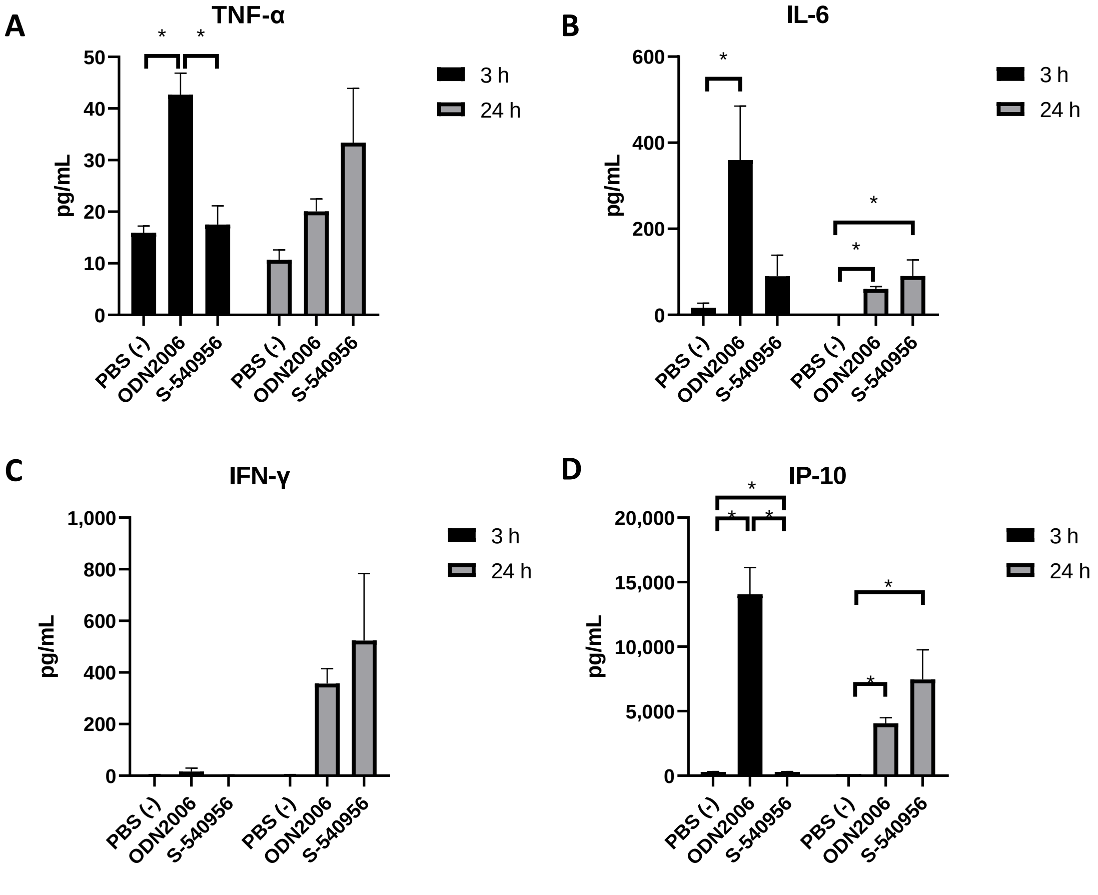

Supplementary Figure 3. Systemic proinflammatory cytokine responses after injection
Mice were intramuscularly injected with S-540956 or ODN2006 (N = 5). TNF-α, IL-6, IFN-γ, and IP-10 levels were measured at 3 and 24 h after injection. The data shown indicate the mean ± SE. $*P < 0.05$, as determined by one-way ANOVA. Data are representative of two independent experiments.

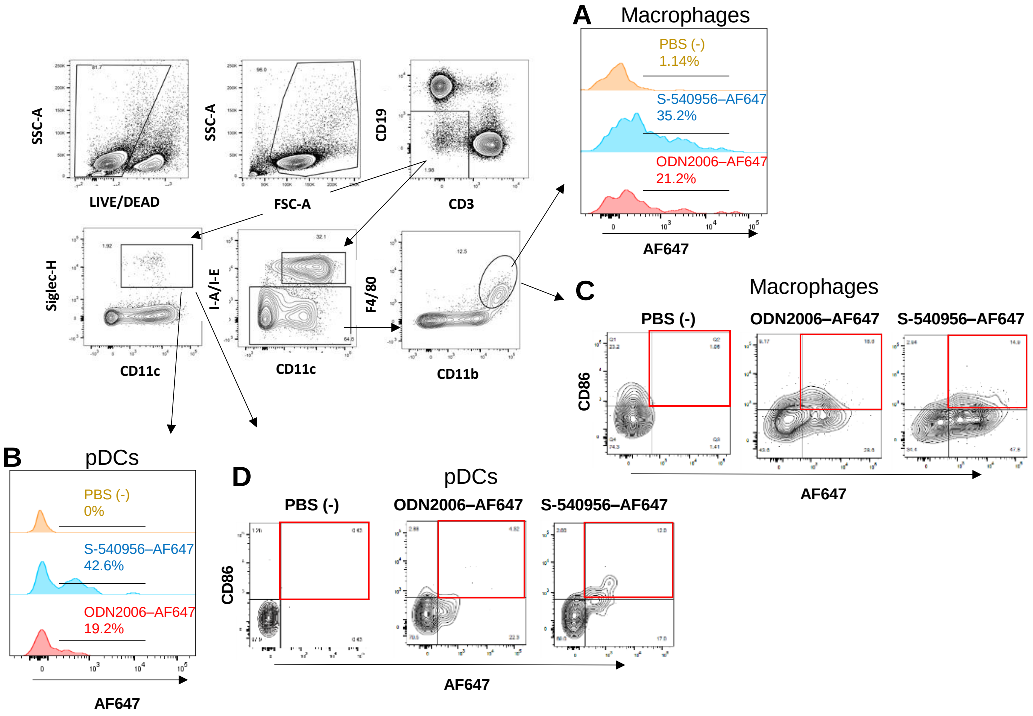

Supplementary Figure 4. A gating strategy to identify macrophages and pDCs. macrophages and pDCs in draining LNs were detected using antibodies that recognize CD3, CD11b, CD11c, I-A/I-E, Siglec-H, and F4/80. The representative data indicate the incorporation of S-540956–AF647 or ODN2006–AF647 by (A) CD11b^{+} F4/80^{+} cells (macrophages) and (B) CD11b^{+} Siglec-H^{+} cells (pDCs) in the draining LNs from samples collected at 4 h after the intramuscular injection. (C) AF647^{+} CD86^{+} CD11b^{+} F4/80^{+} cells and (D) AF647^{+} CD86^{+} CD11b^{+} Siglec-H^{+} cells in the draining LNs were analyzed by flow cytometric analysis at 18 h after the intramuscular injection.

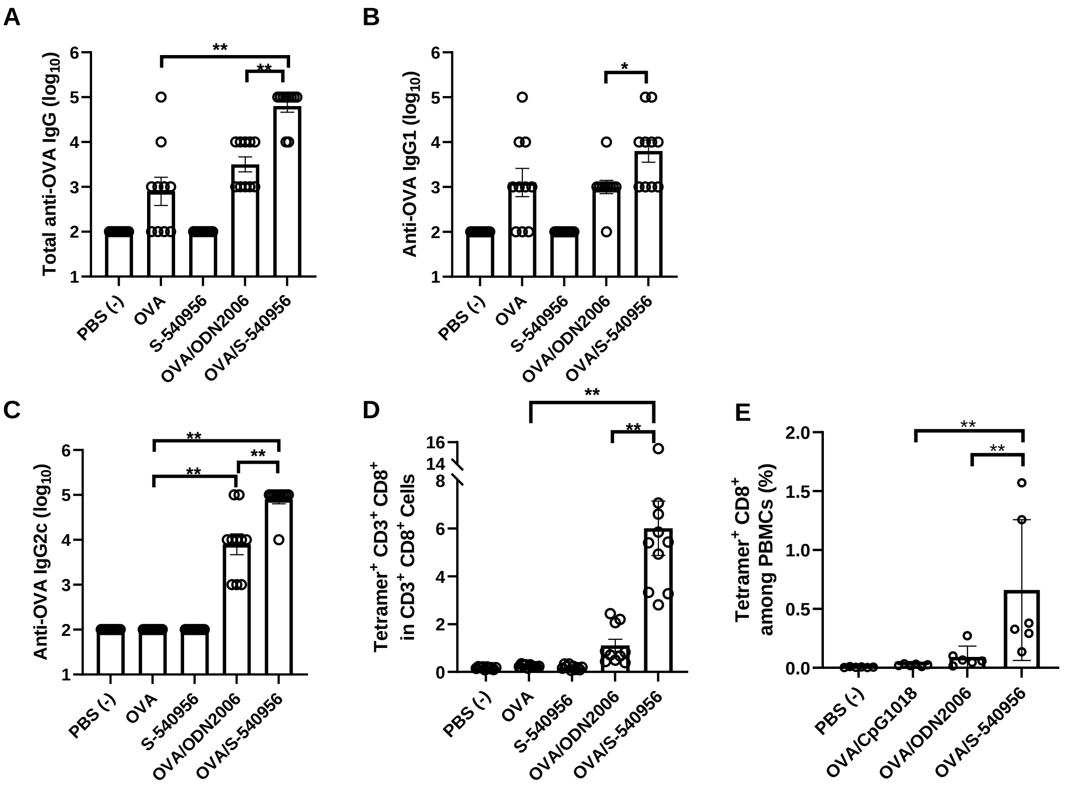

Supplementary Figure 5. S-540956 enhanced humoral and cellular responses to recombinant OVA antigen. Mice were intramuscularly immunized with OVA, S-540956, OVA/ODN2006, or OVA/S-540956 on days 0 and 14 after the first immunization (N = 10). (A) OVA-specific total IgG, (B) IgG1, and (C) IgG2c responses and (D) $CD8^+$ T cell responses were evaluated on day 21 after the first immunization. Mice were intramuscularly immunized with OVA/CpG1018, OVA/ODN2006, or OVA/S-540956 on days 0 and 14 after the first immunization (N = 6). $CD8^+$ T cell responses were evaluated on day 21 after the first immunization. The data indicate the mean ± SE. *$P < 0.05$, **$P < 0.01$, as determined by one-way ANOVA. Data are representative of two independent experiments.

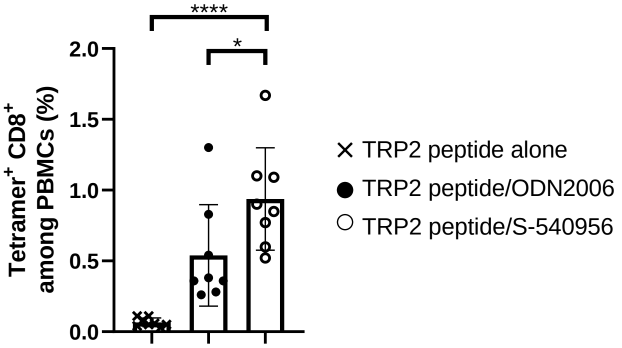

Supplementary Figure 6. Adjuvant effect of S-540956 with a cancer peptide vaccine in mice. Mice were subcutaneously immunized with TRP2 peptide alone, TRP2 peptide/ODN2006, or TRP2/S-540956 on days 0 and 7 after the first immunization (N = 8). Peptide-specific $CD8^+$ T cells among

peripheral blood mononuclear cells (PBMCs) in PBMCs were analyzed by flow cytometry on day 14. The data shown indicate the mean ± SE. *P < 0.05, ****P < 0.001, as determined by one-way ANOVA. Data are representative of two independent experiments.

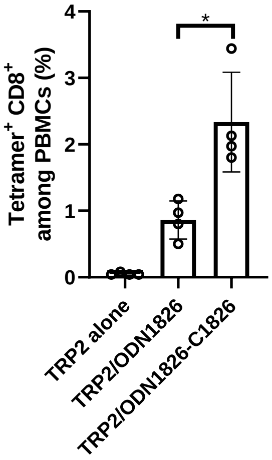

Supplementary Figure 7. The complementary strand with the amphiphilic chain unit enhanced MHC class-I-restricted TRP2 cancer peptide-specific $CD8^+$ T cell induction by ODN1826. Mice were intramuscularly immunized with TRP2 alone, TRP2/ODN1826, or TRP2/ODN1826-C1826 (ODN1826 annealed with the complementary strand with the amphiphilic chain unit) on days 0 and 7 (N = 4). TRP2-specific $CD8^+$ T cells among PBMCs were analyzed by flow cytometry on day 14. The data shown indicate the mean ± SE. The bar graphs indicate the mean ± SE. *P < 0.05, as determined by one-way ANOVA. Data are representative of two independent experiments.

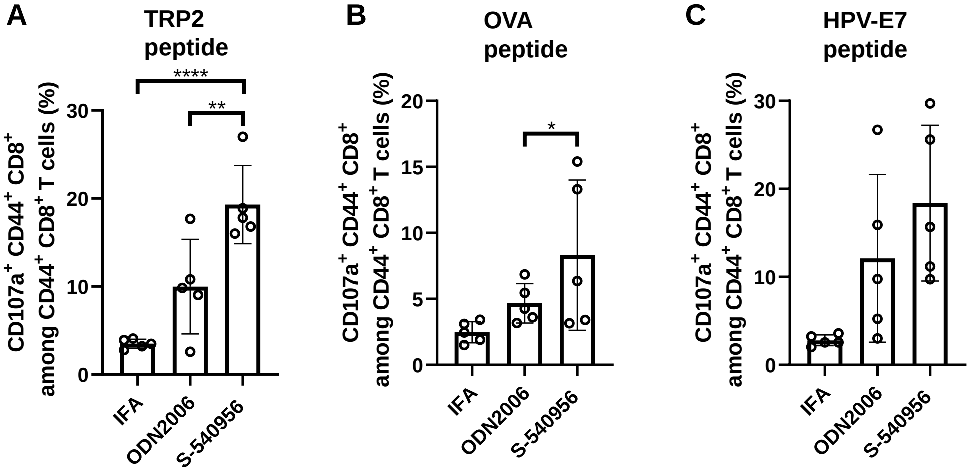

Supplementary Figure 8. S-540956 enhanced MHC class-I-restricted cancer peptide-specific $CD107a^+$ $CD8^+$ T cell responses. Mice were intramuscularly immunized with peptide/S-540956, ODN2006, or Montanide ISA-51 on days 0 and 7 (N = 5). TRP2 (A), OVA (B), or HPV-E7 (C)

peptide-specific CD107a$^+$ CD44$^+$ CD8$^+$ T cells among CD44$^+$ CD8$^+$ T cells in splenocytes were analyzed by flow cytometry on day 14. The data shown indicate the mean ± SE. *$P < 0.05$, **$P < 0.01$, ****$P < 0.001$, as determined by one-way ANOVA. Data are representative of two independent experiments.

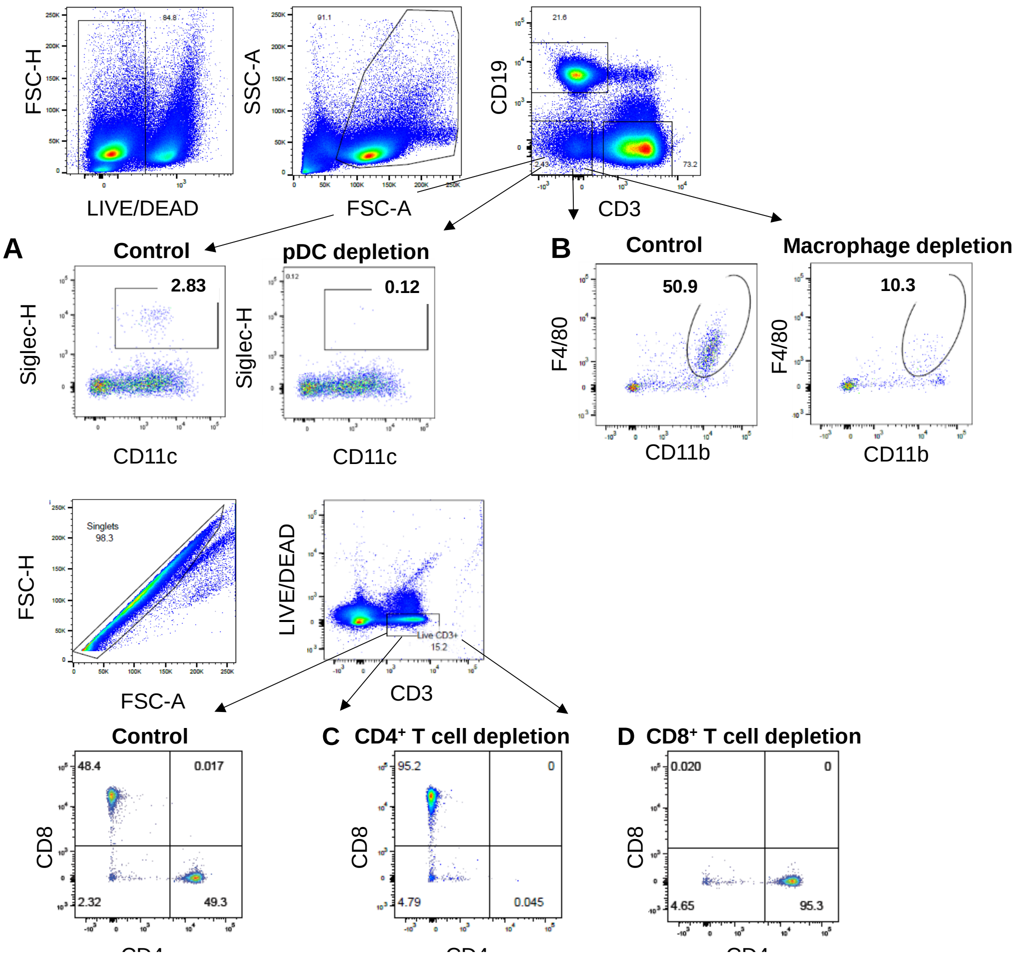

Supplementary Figure 9. A gating strategy to confirm depletions of each cell populations. (A) pDCs depleted by anti-PDCA1 antibody, (B) macrophages depleted by Clophosome-A, (C) CD4$^+$ T cells depleted by anti-CD4 antibody, and (D) CD8$^+$ T cells depleted by anti-CD8α antibody are shown. All samples were collected at 18 h after injection of Clophosome-A or each antibody. Macrophages and pDCs in draining LNs or CD4$^+$ T cells and CD8$^+$ T cells in PBMCs were analyzed by a flow cytometer.
